# BACnet Application Layer over Bluetooth—Implementation and Validation

**DOI:** 10.3390/s21020538

**Published:** 2021-01-13

**Authors:** Nicoleta Cristina Gaitan, Ioan Ungurean

**Affiliations:** 1 Faculty of Electrical Engineering and Computer Science, Stefan cel Mare University of Suceava, 720229 Suceava, Romania; 2 MANSiD Integrated Center, Stefan cel Mare University, 720229 Suceava, Romania

**Keywords:** Internet of Things, Bluetooth, BACnet, smart building

## Abstract

The development of the smart building concept and building automation field is based on the exponential evolution of monitoring and control technologies. Residents of the smart building must interact with the monitoring and control system. A widely used method is specific applications executed on smartphones, tablets, and PCs with Bluetooth connection to the building control system. At this time, smartphones are increasingly used in everyday life for payments, reading newspapers, monitoring activity, and interacting with smart homes. The devices used to build the control system are interconnected through a specific network, one of the most widespread being the Building Automation and Control Network (BACnet) network. Here, we propose the use of the BACnet Application Layer over Bluetooth. We present a proposal of a concept and a practical implementation that can be used to test and validate the operation of the BACnet Application Layer over Bluetooth.

## 1. Introduction

The smart buildings [1] concept is nowadays becoming more popular. The aim of smart buildings is to improve the comfort of the residents in their daily life. This is possible by using gadgets that can assist residents in daily activities, such as controlling and monitoring the lighting system, heating system, utility consumption, etc. In order to respond to this desideratum, it is necessary to integrate monitoring and control systems [2] in the buildings or houses. These systems are known as Building Automation Systems (BASs). BAS systems offer various facilities for interaction with the resident people. In addition to these facilities, Internet of Things (IoT) [3,4] platforms can be developed in order to enable the interaction between people and these monitoring and control systems in a virtual environment. The monitoring and control devices used in smart homes are interconnected throughout communication networks (fieldbuses) based on standardized protocols [5], such as Building Automation and Control Networks (BACnets) [6], LonWorks [5], Modbus [7], and EIB/KNX [8]. The use of these standardized communication networks ensures interoperability between devices provided by different manufacturers. In addition, hardware solutions are being developed to meet the requirements that these devices must comply with, such as predictable event response time [9]. Based on these communication systems, different building monitoring and control software systems can be developed by integrating a wide variety of communication middleware technologies such as the one presented in [10].

In addition, BAS systems that are integrated into smart buildings can be used to provide the support needed for the remote monitoring and/or the self-monitoring of patients with various chronic diseases [11]. At this time, there are multitudes of devices that can remotely monitor various medical parameters [12], methods for the automatic processing [13] of these parameters, and alarms triggering when anomalies are detected [14].

Recently, mobile devices represented by smartphones and tablets have experienced explosive development. They are optimized for more computing power at lower energy consumption and a lower price compared to desktop systems, in addition to their mobility function. Now, the number of these mobile devices has exceeded the number of personal computers [15,16]. Mobile devices, such as smartphones and tablets, are used in daily activities for payments, Internet banking, reading newspapers, etc. These devices have Wi-Fi and Bluetooth communication interfaces. They can also be used to monitor and control smart homes by connecting to the Building Automation System. We propose the use of the BACnet Application Layer over Bluetooth. In order to validate this concept, a test system is developed to demonstrate the functionality of the BACnet Application Layer over Bluetooth. This solution has the advantage that the connection to a BACnet network can be made from any computing device represented by smartphones, tablets, and personal computers.

The following sections are structured as follows: Section 2 contains a short presentation of the BACnet protocol. Section 3 presents BACnet gateways to other protocols presented in the specialized literature, and Section 4 presents the proposed concept related to the implementation of the BACnet Application Layer over Bluetooth. Section 5 presents a practical implementation of the proposed concept. Conclusions are drawn in Section 6.

## 2. BACnet

**Figure 1:**
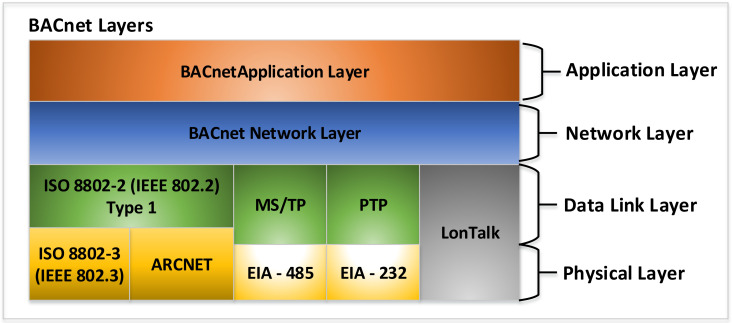
Layers of the Building Automation and Control Network (BACnet) protocol [17].

BACnet is a communication protocol [17] used in the development of smart homes or in building automation. BACnet is organized into four layers, as shown in Figure 1.

At the Application Layer, BACnet is object-oriented. A BACnet device is a collection of objects that contain device-specific features. At the moment, 60 types of objects are standardized [18], the most used types of objects being: Analog Input, Analog Output, Analog Value, Binary Input, Binary Output, Binary Value, Command, and Device. Each type of object has a set of properties such as identifier, name, type, and value. Each property is characterized by a data type and a conformance code that indicates whether the property is mandatory or optional. A BACnet device is characterized by a device object and may contain one, more, or none of the other object types.

The BACnet Data Link Layer organizes/segments data into frames or packets called Network Protocol Data Unit (NPDU) packets that are transmitted to lower levels. The maximum size of a NPDU is determined by the communication technology used at the physical layer. The lower layers are the Data Link and Physical Layers. As can be seen from Figure 1, at lower layers, we can have the following options: Logical Link Control and Ethernet, Logical Link Control and ARCNET, Master–Slave/Token-Passing (MS/TP) and EIA-485, Point-to-Point and EIA-232, and LonTalk.

## 3. Related Works

This section presents BACnet gateway solutions to other protocols and examples of using the BACnet network for smart buildings. We will focus on functionality and how to interact with human operators. We will also present some cases of using Bluetooth technology for the development of smart buildings. They are all on the same topic as the solution presented in this article.

The automation field has developed considerably and uses new technologies such as intelligent equipment, advanced sensors and controllers, and low energy consumption technologies, all being integrated via communication interfaces and appropriate software in order to manage specific operations such as those from the automotive industry, healthcare, or modern buildings. Based on these premises, a research paper [19] proposed the development, design, and experimental validation of a wireless sensor network but also of a web-based platform for monitoring and managing the microgrid of the Renewable Energy Area National Institute for Aerospace Technology (INTA) (Huelva, Spain) [19]. The authors also proposed a solution for managing renewable energy microgrids that can be optimized by reducing energy consumption integrated into an IoT platform with high data processing and storage capacity.

Another research paper [20] proposed solutions on cybersecurity and interoperability for intelligently Building Automation Systems identified in the field of the industrial control system, and as an example, the functions and tools of the BACnet protocol were given. Modernizing old buildings is a real challenge requiring high-cost improvements for modernization, automation, and low energy consumption, and one of the critical tasks in the development of modern urbanized communities relies on smart cybersecurity for building automation.

In [21], a BACnet-ZigBee smart grid gateway is proposed that can be used in smart buildings. This gateway is used to interconnect a BACnet network with a ZigBee network, and the study [21] focused on the establishment of the demand response. In order to perform this analysis, a load control and metering device for the smart grid was implemented, which communicates via ZigBee with the BACnet-ZigBee gateway. The authors concluded that the BACnet-ZigBee gateway and the load control and metering device developed in this study can be used in smart buildings for the development demand response for smart grids. In [22], the authors proposed a BACnet–EnOcean smart grid gateway. Again, the authors proposed a load control and metering device that communicates with the BACnet–EnOcean gateway through EnOcean, and the demand response functionality in buildings was analyzed. Communication delay in the context of complying with the real-time requirements of demand response services in buildings was analyzed.

In [23], the authors proposed a system for multiprotocol parsing for smart buildings. The system works for Modbus TCP/IP, KNX, and BACnet/IP protocols and publishes data purchased from these networks in a unitary manner. A protocol conversion gateway solution for fieldbuses used in smart buildings is presented in [24]. The gateway is developed on an embedded system with the Sitara AM335X microprocessor provided by Texas Instruments and integrates Modbus/RTU, BACnet/MSTP, and DL/T645-2007 protocols. These protocols use RS485 as a line protocol and are converted to the Modbus/TCP protocol. The authors concluded that the proposed solution achieves satisfactory performance in real time and in terms of stability. An Internet of Things system design and development based on Bluetooth Low Energy (BLE) is proposed in [25]. In this study, the authors present BLE features that can be used to build an Internet of Things system, they propose a novel hardware for BLE and provide ports for the open-source Contiki operating system.

A compressive study related to the environmental monitoring technologies used in smart buildings is presented in [26]. In terms of communication technologies, this study identifies wireless technologies such as ZigBee, Wi-Fi, Bluetooth, 6LoWPAN, LoRaWAN, EnOcean, and wired technologies such as Ethernet, Modbus, and BACnet that can be used for the development of environmental monitoring systems. The study describes in detail Bluetooth and Bluetooth Low Energy (BLE) technologies as common wireless technologies used in smart buildings. The authors also study the sensors that can be used for environmental monitoring.

## 4. The Concept Related to the Implementation of the BACnet Application Layer over Bluetooth

**Figure 2:**
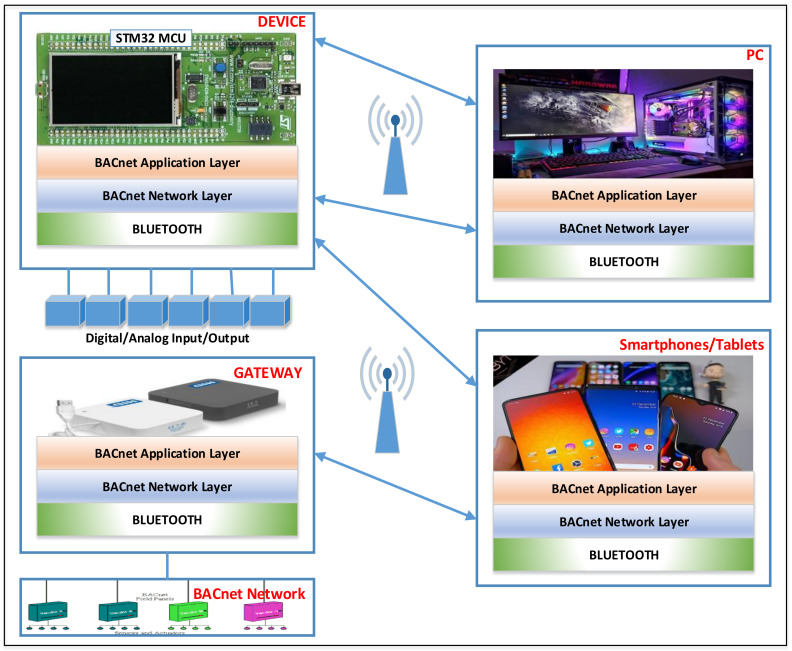
The proposed concept for the implementation of the BACnet Application Layer over Bluetooth.

We propose the implementation of the BACnet Application Layer over Bluetooth. The proposed concept allows us to define BACnet objects over Bluetooth, taking advantage of standardizing definitions of the data format that is transmitted over Bluetooth. This concept allows BACnet networks to be available via Bluetooth, and smart BACnet devices with Bluetooth connectivity can also be designed and developed. Figure 2 presents the proposed concept. Thus, it is possible to design and develop a device for smart homes with digital/analog inputs/outputs, sensors, and actuators that implement the BACnet Application Layer over Bluetooth. 

Software applications that are connected by this device and can monitor and control the device can be used on smartphones/tablets. It does not require a Wi-Fi network or an Internet connection; it requires only a smartphone/tablet with a Bluetooth interface (all modern smartphones are equipped with a Bluetooth interface). There may also be a similar PC application that can run on PCs, laptops, or tablets with the Windows operating system and Bluetooth connection. Additionally, for the integration of traditional wired BACnet devices, a gateway that connects to the BACnet network and translates data over a Bluetooth connection can be designed and developed. This allows monitoring and controlling the classic BACnet devices via the application on smartphones/tablets/PCs and Bluetooth. Figure 2 illustrates this concept by presenting a BACnet device with a Bluetooth connection and presenting a gateway that connects to a classical BACnet network. The functionalities of these two devices can be integrated into a single device that functions as a BACnet device over Bluetooth and as a gateway for a classical wired BACnet network. This is useful to counteract the disadvantage of point-to-point Bluetooth communication and the use of the advantages of Bluetooth communication that can be used on mobile smartphones that are widespread.

Figure 2 presents only the concept; the implementation can be done in several ways. It should also be borne in mind that Bluetooth is a very convenient type of communication; however, it can only be done point by point, and there is no possibility to make multiple connections to the BACnet device implemented over Bluetooth. This is the main disadvantage of the proposed solution. The next section presents the implementation of this concept to validate the operation of the BACnet Application Layer over Bluetooth.

**Figure 3:**
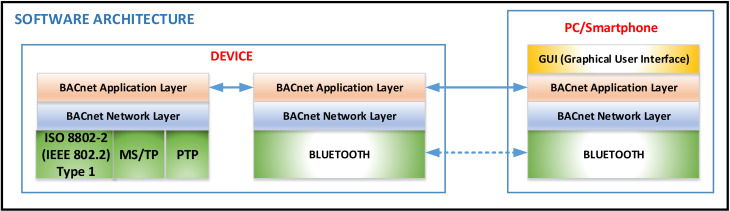
The software architecture of the proposed concept.

Figure 3 presents the software architecture of the proposed concept. In this figure, we can see the main software components of the proposed solution. Thus, on the PC/smartphone is implemented a BACnet stack that uses the transport layer of Bluetooth technology. A graphical interface is included over this stack that allows us to view the data received from the BACnet device and send the data to the BACnet device. This is actually a BACnet-explorer-type application. A BACnet stack is implemented on the BACnet device using the transport layer of the Bluetooth technology. In this case, the device will operate as a BACnet device; the only difference is that it uses another technology at the transport layer (such as the Device from Figure 2). In addition to this stack, a BACnet stack can be implemented on the device, which uses the classic technologies for the transport layer. Therefore, the device can function as a gateway to classic BACnet networks (such as the Gateway from Figure 2). The connection between PC and device is made at BACnet Application Layer from a logical point of view and at the Bluetooth layer from the physical point of view.

One advantage of the proposed solution is that we can use smartphones or tablets to connect to the BACnet network. This allows us to monitor and graphically display the parameters and send commands to the BACnet network by taking advantage of the mobility capabilities provided by smartphones and tablets. Another advantage is the convenience of using smartphones and tablets. The main disadvantage is that Bluetooth communication can be used over short distances [27] (usually up to 10 m, with a maximum range of 100 m without obstacles) and is not stable enough to perform real-time control of smart building functionality, such as demand response. 

## 5. Concept Implementation and Validation

In order to validate the proposed concept, we implemented a test system that aims to exemplify how communication is achieved at the BACnet Application Layer over Bluetooth. Since the functionality of the other layers of the BACnet model is similar to other communication protocols, it was chosen to focus on how it works at the Application Layer. The test system is divided into two components. The first component is a software application that is loaded on the STM32F4DISCOVERY development board for building a BACnet device and communication via Bluetooth using an HC-05 Bluetooth module. The second component is the graphical software application for PC that aims to highlight the functionality of the BACnet services via Bluetooth. The BACnet device implements one Device object and two Analog Input objects. The implemented services are the following: Who-Has, I-Have, Who-Is, I-am, ReadProperty, ReadPropertyConditional, ReadPropertyMultiple, WriteProperty, WritePropertyMultiple, AddListElement, RemoveListElement, CreateObject, and DeleteObject. 

**Figure 4:**
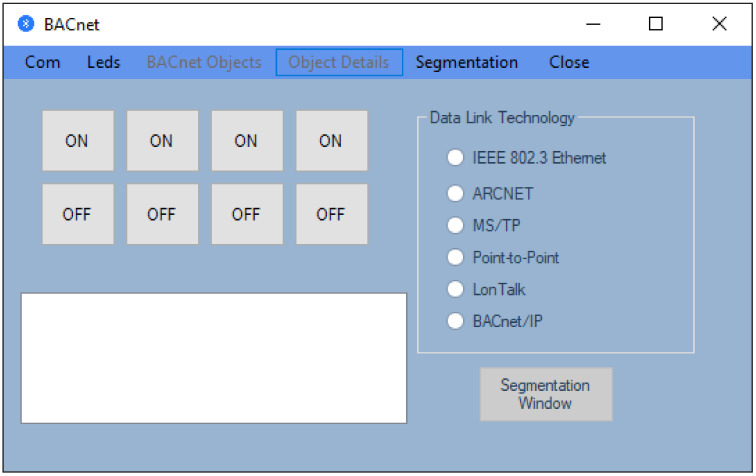
The main window of the application executed on a PC.

The first part of the test system is developed and implemented using the KEIL Microcontroller Development Kit (MDK) debugging and tracing environment. With the help of this toolset, a virtual BACnet device is created that contains three virtual objects: an object of the Device type and two objects of the Analog Input type. This application also takes into account the fact that the data will be transmitted using a serial port. For this reason, in the application, a software module is used that allows the selection of data according to the request received throughout the Universal Asynchronous Receiver/Transmitter (UART) port via the HC-05 Bluetooth module, the request that is transmitted from the graphics application. The second part of the test system is a graphical application developed to run on PCs with the Windows operating system. The main window of this application is shown in Figure 4.

**Figure 5:**
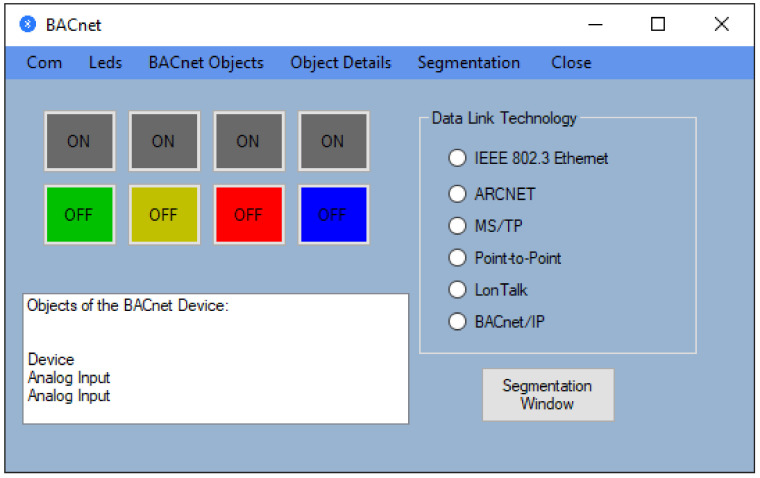
The BACnet objects provided by the device.

As can be seen in Figure 4, the main window of the graphical application has a submenu that is disabled; the Segmentation option from the menu, which has the role to execute an interface that computes the number of segments; and the Close option from the menu, which closes the application. In the beginning, we presented the graphical interface of the application that communicates with the BACnet device implemented on the STM32F4DISCOVERY development board. In the first phase, from the Com option, by selecting the Search suboption, we must select a COM port because the Bluetooth device is seen by the PC as a virtual COM port. This virtual port is configured in the Bluetooth connection setup stage between the PC and the Bluetooth device. At this step, we know the COM port number associated with the BACnet device, and the next step is to select this port and select the Connect suboption from the Com option to activate the connection between the application and the BACnet device via Bluetooth wireless communication. If the steps are correctly followed, the window shown in Figure 5 will be obtained (the display box on the bottom left contains the message: “The connection was activated successfully.”).

Once we obtain the window shown in Figure 5, we can see that we have access to the LED option (to the LEDs) that allows us to turn on and turn off the LEDs on the development board. At the same time, we have access to the “BACnet Objects” option from the menu that, after accessing, will display objects available on the device BACnet, as shown in Figure 5. This option uses the Who-Has service in order to display all the objects of the BACnet device implemented on the STM32F4DISCOVERY development board.

**Figure 6:**
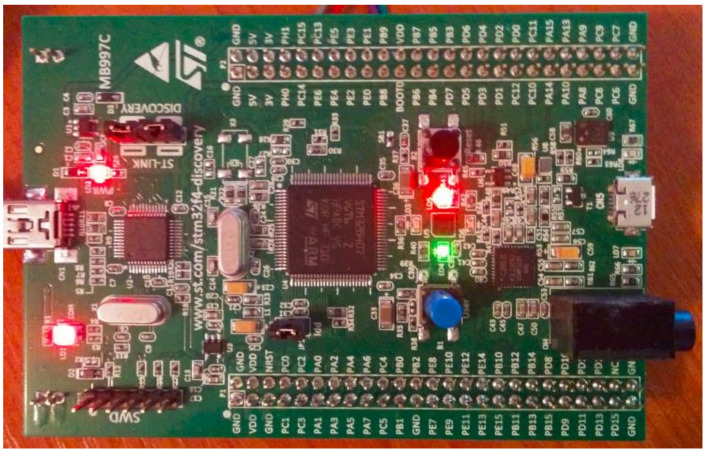
The requests indicated by LEDs on the STM32F4DISCOVERY development board.

At the same time, by selecting the “BACnet Objects” option, the board will indicate that this option was chosen by turning on the red and green LED, as can be seen in Figure 6.

In order to view the properties of an object, the object to be analyzed must be selected from the selection list from the bottom left, according to Figure 5, followed by the selection of the “Object Details” option. If a selection is made that proves invalid, then a warning message will be displayed regarding the problem.

**Figure 7:**
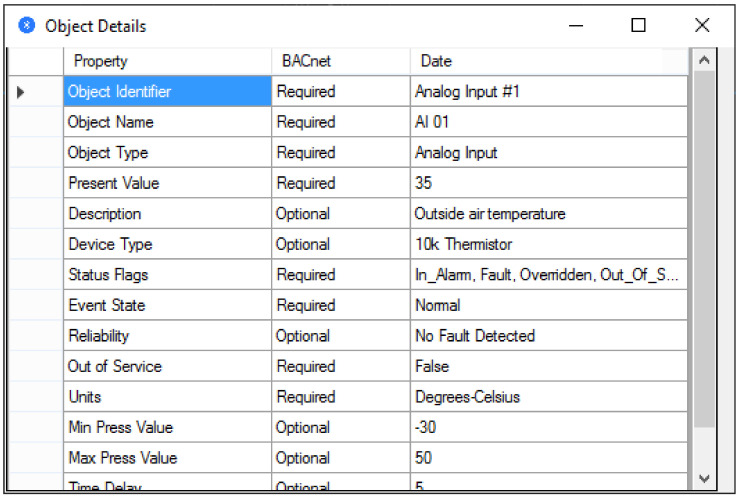
Details related to a BACnet object.

After selecting the object type and the specific option, a new window will appear showing the properties of the selected object as shown in Figure 7. Consequently, the properties of the objects within the BACnet device are obtained, which is essential for the protocol because this information is at the base of communication in a BACnet network. One of the properties is the value associated with the object. This exemplified the interaction between objects built on a BACnet device and services that create requests for information embedded in objects.

**Figure 8:**
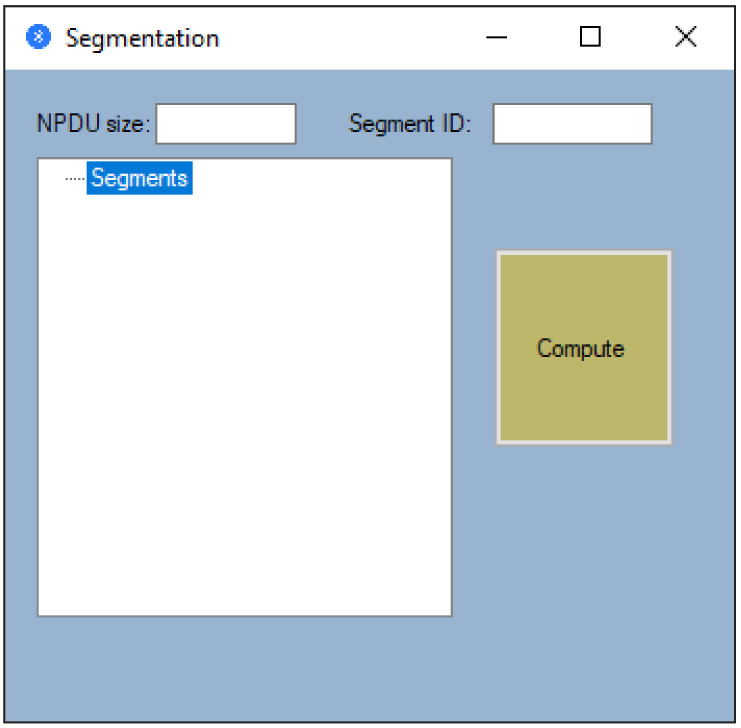
Segmentation window.

Furthermore, segmentation takes place at the Network Layer (Bluetooth from Figure 2), and in this sense, a computer was introduced in the application that has the role of determining the number of segments for a certain transmission technology and the information included in each segment. If we refer to the main window presented in Figure 4, we will notice that there is an option called Segmentation). After selecting this option, we will be allowed to choose the technology at the Data Link layer as it is exemplified in Figure 8. If no Data Link technology is selected, and if we want to open the segmentation window, then a warning message will be displayed.

**Figure 9:**
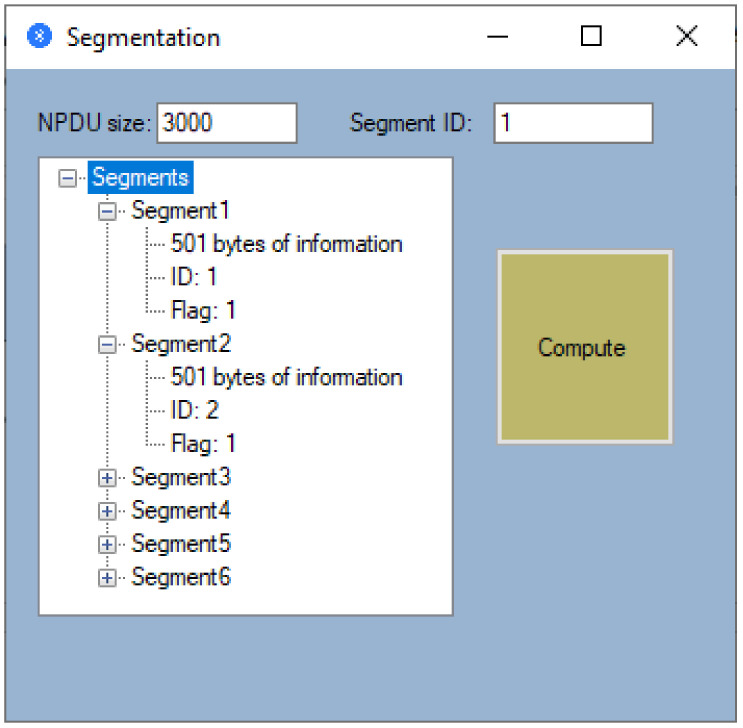
Example of a segmentation operation.

In order to calculate the number of segments, it is absolutely necessary to enter the size of the Network Protocol Data Unit (NPDU), which has at least 2 bytes given by the segment header and the Identification (ID) from which the segment numbering starts. After entering this data, we can proceed to calculate the number of segments. Thus, for the Point-to-Point (Bluetooth) technology previously selected, and for an NPDU of 3000 bytes that has the ID of the first segment equal to one, a number of six segments were obtained (see Figure 9). As can be seen in Figure 9, segmentation is performed according to the Data Link technology used. Thus, in this case, the first 5 segments carry the maximum information given by 501 bytes, and in addition to the transported information, the segments are characterized by an ID and a Flag that takes the 0 value if the transmitted segment is the last.

The security vulnerabilities of the proposed solution are generated by Bluetooth communication. A detailed analysis of these security vulnerabilities is presented in [28]. To address these issues, Bluetooth communication is implemented in Security Mode 4 that uses the encrypted key exchange.

## 6. Conclusions

Herein, we proposed and tried to present the use and the utility of the BACnet Application Layer over Bluetooth. This can be performed by designing and developing a BACnet device with a Bluetooth connection or by implementing a gateway that connects to a classic BACnet network via Bluetooth. We proposed and validated a concept by implementing a BACnet device on the STM32F4DISCOVERY development board, which contains an HC-05 module for Bluetooth connection and a PC application that communicates with this module via Bluetooth, all being implemented at the BACnet Application Layer over Bluetooth. The proposed solution is convenient because most modern smartphones and tablets support Bluetooth communication, the main disadvantage being that Bluetooth allows point-to-point connection only. In fact, the purpose of the proposed solution is to allow access to the BACnet network for the visualization of the measured parameters (temperature, humidity, light intensity, energy consumption, etc.) and to transmit simple commands such as turning on/off the light and not to perform real-time control of the smart building.

## Data Availability

Data sharing not applicable.
